# Subgroup Preference Neural Network

**DOI:** 10.3390/s21186104

**Published:** 2021-09-12

**Authors:** Ayman Elgharabawy, Mukesh Prasad, Chin-Teng Lin

**Affiliations:** Australian Artificial Intelligence Institute, School of Computer Science, University of Technology Sydney, Ultimo, Sydney 2007, Australia; ayman.elgharabawy@uts.edu.au (A.E.); Chin-Teng.Lin@uts.edu.au (C.-T.L.)

**Keywords:** preference learning, neural network, label ranking, stairstep, spearman rank correlation

## Abstract

Subgroup label ranking aims to rank groups of labels using a single ranking model, is a new problem faced in preference learning. This paper introduces the Subgroup Preference Neural Network (*SGPNN*) that combines multiple networks have different activation function, learning rate, and output layer into one artificial neural network (*ANN*) to discover the hidden relation between the subgroups’ multi-labels. The *SGPNN* is a feedforward (*FF*), partially connected network that has a single middle layer and uses stairstep (*SS*) multi-valued activation function to enhance the prediction’s probability and accelerate the ranking convergence. The novel structure of the proposed *SGPNN* consists of a multi-activation function neuron (*MAFN*) in the middle layer to rank each subgroup independently. The *SGPNN* uses gradient ascent to maximize the Spearman ranking correlation between the groups of labels. Each label is represented by an output neuron that has a single *SS* function. The proposed *SGPNN* using conjoint dataset outperforms the other label ranking methods which uses each dataset individually. The proposed *SGPNN* achieves an average accuracy of 91.4% using the conjoint dataset compared to supervised clustering, decision tree, multilayer perceptron label ranking and label ranking forests that achieve an average accuracy of 60%, 84.8%, 69.2% and 73%, respectively, using the individual dataset.

## 1. Introduction

Preference learning (*PL*) is an extended paradigm in machine learning that induces predictive ranking models from experimental data [1,2,3]. *PL* is applied to many different research areas such as knowledge discovery and recommender systems for learning the ranking [4]. Objects, instances, and label ranking are the three main categories of *PL*. Label ranking (*LR*) is a challenging problem that has gained importance in information retrieval by search engines [5,6]. Unlike the common problems of regression and classification, label ranking involves predicting the relationship between multiple label orders. Multi-label ranking problems are based on preference relations over a permutation space ω where each member of a group of *k* labels has a preference λ value, $L=\{\lambda_{1},\lambda_{2},...,\lambda_{k}\}$, where the differences of λ value represent preference relations (≻,⪰,⊁,⋡,∼,≺,⪯) [1,7]. However, real-world data can be ambiguous and often lack preference relations between two or more labels, and the missing relations can be mapped to an indifference ∼, or incomparability ⊥, relation [8,9]. These two relations create a partial order on the ω space where $\lambda_{a}⊥\lambda_{b}$ or $\lambda_{a}∼\lambda_{b}$. The partial relations are solved in terms of the relation between labels in one ω space in [10,11]. For example, $\pi =(\lambda_{a}≻\lambda_{b}∼\lambda_{c}≻\lambda_{d})$ is mapped to π=(1,2,2,3) and $\pi =(\lambda_{a}≻\lambda_{b}≻\lambda_{c}⊥\lambda_{d})$ is mapped to π=(1,2,3,0). However, sometimes the data collected from the likes of recommender systems, elections, and surveys deviate from the population and in such cases label ranking cannot be predicted using the same learning model. Such a deviation is addressed by extracting patterns to identify the subgroup of data for the interesting targets using subgroup discovery (*SD*) approaches [12]. Subgroup discovery (*SD*) is descriptive induction data mining technique that discovers interesting associations among different variables with respect to a property of interest in many fields [13,14]. i.e., the relation of incidence of acute kidney injury (AKI) in patients with COVID-19 [15]. Existing *SD* utilises different methodologies for searching, pruning, and ranking subgroups [16]. Leeper, T. introduced conjoint analysis on subgroup preferences in the study of political preferences to give better interpretations and average marginal component effects [17]. In decision making, combining the weights using different weight calculation methods into a single set of weights is introduced by Deepa, N. [18]. The weights express the criteria and play an essential role in making correct decisions. Cheng, C. [19] used *SD* to validate the restricted classification culture value schemes of prevalence social media addictions. The approach of collecting the data from multiple sources processed by an expert system to be classified by *MLP* is proposed by Vincent, D. [20] to evaluate agricultural lands suitability.

Preference mining (*PM*) is an extended domain of *PL* and *SD*, which aims to discover the local patterns and deviations of subsets of data [21,22]. Using conjoint model based on the fusion of a different group of data’s sensors has been introduced in emotion recognition by Pandeya, Y. [23]. It uses the deep learning to classify the emotions [23] from audio and video information. Rueping, S. proposed subgroup ranking using the support vector machine (SVM) to rank subgroups with respect to the user’s concept of interestingness [24].

The Label ranking takes one of the following two form of restrictions.

- Restricted label order π = ($\lambda_{a}≻\lambda_{b}≻\lambda_{c}≻\lambda_{d}$) can be represented as π=(1,2,3,4).
- Non-restricted total order π = ($\lambda_{a}≻\lambda_{b}≃\lambda_{c}≻\lambda_{d}$) can be represented as π=(1,2,2,3), where *a*, *b*, *c* and *d* are the label indexes and $\lambda_{a}$, $\lambda_{b}$, $\lambda_{c}$ and $\lambda_{d}$ are the ranking values of these labels respectively.

The pairwise approach was first introduced by Hüllermeier, E. [25] to divide the label ranking problem into several binary classification problems in order to predict the pairs of labels, i.e., $\lambda_{i}≻\lambda_{j}$ or $\lambda_{j}≺\lambda_{i}$ for an input *x*. Cheng, W. and Hühn, J. proposed the instance-based decision tree to rank the labels based on predictive probability models of a decision tree [26]. Grbovic, M. combined both a decision tree and supervised clustering in two approaches for label ranking by mapping between instances and label ranking space [27]. The artificial neural network (*ANN*) for label ranking was first introduced as (RankNet) by Burges, C. to solve the problem of object ranking for sorting web documents from a search engine [28]. RankNet uses the Gradient descent and probabilistic ranking cost function for each object pair. The multilayer perceptron for label ranking (*MLP-LR*) [29] employs a network architecture using a *sigmoid* activation function to calculate the error between the actual and expected values of the output labels. However, it uses a local approach to minimize the individual error per output neuron by subtracting the actual predicted value and using Kendall error as a global approach. However, ranking error function was not used before in backpropagation (*BP*) and learning steps. The ranking methods mentioned above and their variants have some issues that can be broadly categorized into two types:

- The ranking methods are based on probability and classification; thus, They do not learn the preference relation between labels divided into groups.
- The ranking methods learn both unrestricted and restricted ranking labels using the same learning approach.

This paper proposes *SGPNN* as a tool to support the *SD* analysis to rank the discovered subgroup. In addition, *SGPNN* converts unrestricted label ranking to group of restricted labels and learn the groups of labels simultaneously using one model. The *SGPNN* built upon preference neural network (*PNN*) to rank subgroup label data $D\in \{\langle x_{n},\left(\pi_{n1}⊥\pi_{n2}...⊥\pi_{nm}\right)\rangle \}$ where $\pi_{n}$ is a group of labels and m= number of subgroups. The primary motivation of this work is to build a unified predictive ranking model instead of having different models for different labels group.

The labels groups are employed in the following scenarios:

1. Real customer data often explicitly rate different categories of products and services as multi-label subgroups, e.g., restaurant rating based on food quality and customer services [30].
2. Multi-label ranking of related datasets collected in different time periods, e.g., German elections in 2005 and 2009 [31,32].
3. Multi-label data that have unrestricted preference relations between labels are converted into connected subgroups that have restricted relations. This can be seen in the sushi dataset [33,34] where $\lambda_{a}≻\left(\lambda_{b},\lambda_{c}\right)$ is solved by 2 subgroups using the indifference ∼ or incomparable ⊥ relations as $\left(\lambda_{a}≻\lambda_{b}≻\lambda_{c}\right)∼\left(\lambda_{a}≻\lambda_{c}≻\lambda_{b}\right)$ or $\left(\lambda_{a}≻\lambda_{b}≻\lambda_{c}\right)⊥\left(\lambda_{a}≻\lambda_{c}≻\lambda_{b}\right)$. Another example of no ground-truth data where one data record has two labels $\pi_{x}=\left(\lambda_{a}≻\lambda_{b}\right)$ and $\pi_{x}=\left(\lambda_{b}≻\lambda_{a}\right)$ which are mapped to $\pi_{x}=\left(\lambda_{a}≻\lambda_{b}\right)⊥\left(\lambda_{b}≻\lambda_{a}\right)$.

The current challenge of the proposed *SGPNN* is the lack of datasets that represents the labels in a subgroup. Therefore, the datasets are synthesized from real data from single or multiple domains.

To sum up, the key contributions in this paper are:

- Introducing a novel multi activation function neuron (*MAFN*) which uses multiple activation function where each function serve a group of output labels.
- Ranking groups of label has incomparable/indifference relation simultaneously.
- Discovering the hidden relation between different datasets by learning them together in one model is a novel approach to build an accumulative learning approach.
- Solving the data ambiguity by removing the duplicated record which have different labels and marking the class overlap data with subgroup labels.

## 2. The Proposed *SGPNN*

This section gives an overview of the activation function, error functions, *PNN* and *SGPNN* architecture and its functionality.

### 2.1. StairStep (*SS*) Activation Function

The classical *ANN* activation functions have a binary output or range of values based on a threshold. However, these functions do not produce multiple values for different segments of the *x*-axis. The stairstep (*SS*) function is introduced to slow the effective learning rate around different rank values on the *y*-axis to solve the problem of ranking instability. The *SS* function is designed to be non-linear, monotonic, continuous, and differentiable by using a polynomial of *tanh(x)* function. The step width keeps the ranking during the forward and backward process stable.

**Figure 1:**
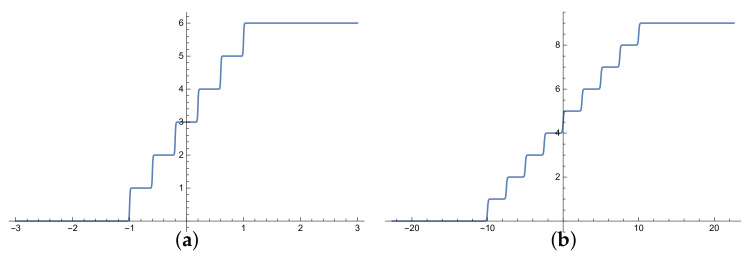
SS activation function (**a**) number of steps n=6 and boundary value b=1. (**b**) number of steps n=9 and boundary value b=10.

Aizenberg, I. [35] proposed a generalized multiple valued neuron using convex shape to support complex numbers neural network and multi-values numbers. In addition, Moraga, C., and Heider, R. [36] introduced a similar function to design networks for realizing any multivalued function; however, Moraga, C. used exponential function derivative did not give promising results in the *PNN* implementation using the ranking objective function in *FF* and backpropagation (*BP*) steps. Each neuron has a multivalued *SS* activation function used to calculate the ranking between labels, s=n+1 where *s* is the number of steps and *n* is the number of ranked labels. The *SS* has a fixed sharp stair-like edge to accelerate the convergence rate and provide multivalued output from −∞ to ∞ as shown in Figure 1. In order to be able to rank a large number of labels, the *SS* function effectively has a dynamic domain (on the *x*-axis), depending on a parameter *b*, to achieve adequate step width on the x-axis. Therefore, the input data are normalized from −b to *b*. We assume a heuristic rule of boundary value to capture the data range as b=2n, where *b* is the geometric *x*-axis boundary.

**Figure 2:**
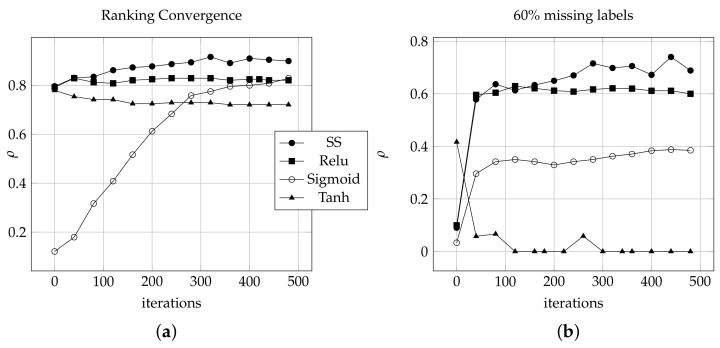
Comparison of activation functions ranking of iris dataset. (**a**) has a complete labels. (**b**) has 60% missing labels.

**Figure 3:**
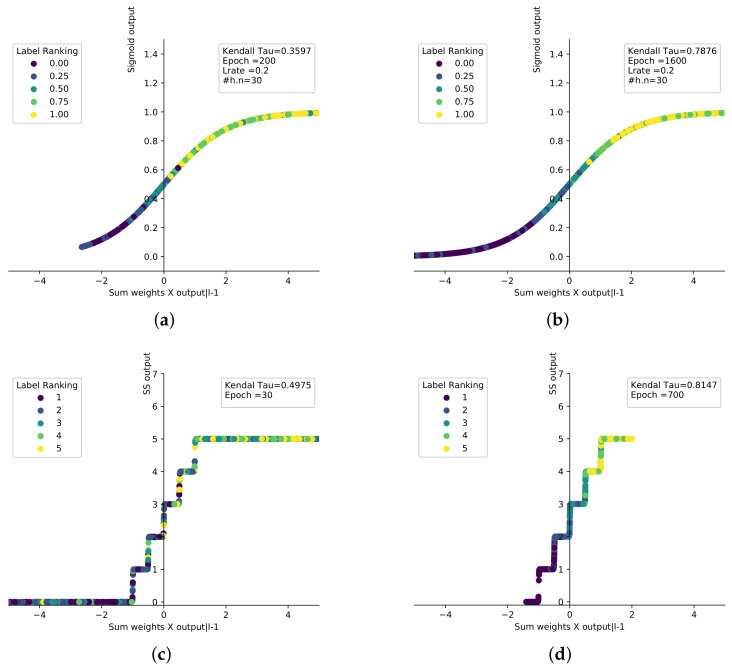
The graphical comparison between convergence of *Sigmoid* and *SS* functions to rank stock dataset, (**a**) *Sigmoid* has τ=0.3597 and epoch = 200. (**b**) *Sigmoid* has τ=0.7876 and epoch = 1600. (**c**) *SS* has τ=0.4975 and epoch = 30. (**d**) *SS* has τ=0.8147 and epoch = 700.

The *SS* activation function is given in Equation (1).

(1) $$\begin{array}{c} f\left(x\right)=-\frac{1}{2}\left(\sum_{i=0}^{n}\tanh\left(\frac{-100}{b}x+c\left(1-\frac{2i}{n-1}\right)\right)\right)+\frac{n}{2} \end{array}$$

where c=100 is a constant value chosen to create the sharp step edge, *n* is the number of ranked labels and *SS* is located between the geometry boundary −b and *b* on the *x*-axis. Each step represents a preference value on the *y*-axis from 1 to ∞. The incomparable relation between labels ⊥ is mapped to 0. As shown in Figure 2, the *SS* step horizontal segments are not an absolutely horizontal line but slope slightly to slow the changing rate around preference values. *SS* has been tested against other activation functions and it shows a ranking performance stability for complete and missing 60% of labels as shown in Figure 2a,b respectively. Figure 3 illustrates the graphical comparison between of *Sigmoid* and *SS* functions to rank stock dataset by summation the output weights for each neuron of middle layer. *Sigmoid* reaches from ρ= 0.3579 in 200 epochs to ρ= 0.7876 in 1600 epochs as shown in Figure 3a,b for ranking 5 labels. However, the *SS* function reaches from ρ= 0.4975 in 30 epochs to ρ= 0.8147 in 700 epochs as showing in Figure 3c,d using the same hyperparameters for ranking 5 labels.

### 2.2. Error Function

Two main error functions have been used to measure the quality of ranking, Kendall’s τ [37], and Spearsman’s ρ [38]. This paper uses Spearman’s ρ to train the *PNN* because Kendall’s τ lacks continuity and differentiability. Spearman’s ρ measures the relative ranking correlation between actual and target ranks, which is also more appropriate than the total square error because a low squared error does not necessarily mean a high ranking correlation between labels. We do not use the absolute difference of the root means square errors (*RMSs*) because the gradient descent may not decrease the ranking error. i.e., $\pi_{1}=\left(1,2.1,2.2\right)$ and $\pi_{2}=\left(1,2.2,2.1\right)$ have a low *rms* of 0.081 but a low ranking correlation ρ=0.5 and τ=0.3. We use the *BP* algorithm to train the *PNN* thus maximizing The Spearsman’s ρ in Equation (2), and its derivative is used as the stopping criteria for the learning process.

(2) $$\rho =1-\frac{6\sum_{i=1}^{n}{\left(y_{i}-yt_{i}\right)}^{2}}{n(n^{2}-1)}$$

where $y_{i}$, $yt_{i}$, *i* and *n* represent rank output value, expected rank value, label index, and number of instances, respectively.

### 2.3. Preference Neural Network (PNN)

#### 2.3.1. One Middle Layer

The preference neural network (*PNN*) is a simple fully connected network with a single hidden layer which provides desirable ranking performance due to the *SS* activation function [39]. We performed experiments on 12 benchmark label ranking datasets [26] which show that increasing the number of hidden layers does not improve the performance, but rather it has adverse effects. This performance declined due to The *SS*’s limited output variation that reduces the degrees of freedom when solving more complex problems. As mention by Lippmann, R. that three layers are sufficient to form arbitrarily complex decisions. [40], However, this is based on the current activation functions that have variations of output comparing to *SS* function.

**Figure 4:**
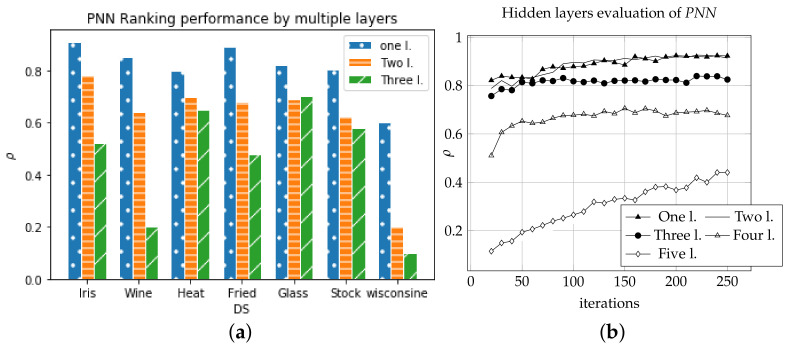
The Number of hidden layers comparison using *PNN* and *SS*. (**a**) Ranking using benchmark datasets [26]. (**b**) Convergence of Average ranking ρ of iris and wine in 200 epochs.

*PNN* experimented using multi-hidden layers using benchmark data at *KEBI* repository [26]. The result showed decreasing ranking correlation by increasing the number of hidden layers, as shown in Figure 4.

#### 2.3.2. Preference Neuron (*PN*)

**Figure 5:**
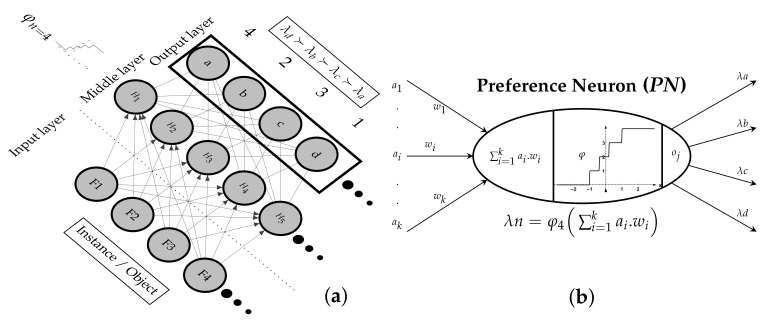
Architecture of Preference Neural Network and Neuron (**a**) Preference Neural Network (**b**) Preference Neuron (*PN*) where $\varphi_{n=4}$, $f_{in}$ = 4.

A preference neuron (*PN*) is a neuron that has an *SS* activation function. The *PN* in the middle layer connects to only *n* output neurons (s=n+1) where *s* is the number of steps and *n* is the number of output ranked labels. The middle and output *PN*s produce a preference value from 0 to ∞ as shown in Figure 5b where *PN* has n=4. The number of output neurons is equal to the number of stair steps, as illustrated in the network architecture Figure 5b. However, the neuron has one output value per epoch, The Figure 5b shows *n* outputs connected to *n* neurons because *SS* has *n* stair steps values as presented in network architecture in Figure 5a.

The *PNN* ranks multi-labels by predicting the preference value for each output neuron by mapping the order to relative ranking around integer values from 1 to ∞ and 0 is mapped to incomparable ⊥ or undifferentiated ∼ relations. Each output neuron represents a label index as shown in Figure 5. i.e., when $L=\{\lambda_{a},\lambda_{b},\lambda_{c},\lambda_{d}\}$ and π=(d≻b≻c≻a), the output neurons will be π=(4,2,3,1) or approximation values that make ρ≃1, i.e.,  π=(3.9,1.8,3.1,0.9) due to *SS* sharp edges. We use gradient ascent to maximize the Spearman ρ. a comparison with conventional *FF-ANN* is shown in Table 1. The architecture simplifies the learning process by eliminating the looping of the hidden layers. The *FF*, *BP*, and updating of weights (*UW*) are executed in two steps. Therefore, the batch weight updating technique does not apply to the *PNN* architecture, and pattern update is used in one step [41]. The network bias is low due to the limited neuron output variation. *PNN* is proposed for one group of label ranking. However, the architecture is not suited to rank different lengths of outputs. To rank different group sizes, a different *SS* function per group is required, which is not provided by the *PNN*.

### 2.4. SGPNN Architecture

This section describes the architecture of *SGPNN* and its functionality.

#### Multi Activation Function Neuron (*MAFN*)

**Figure 6:**
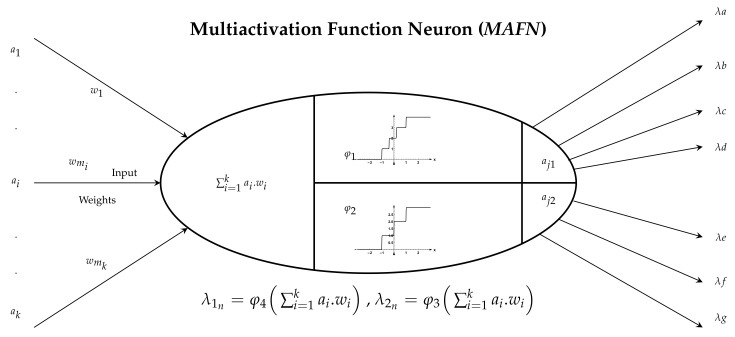
The structure of the *MAFN* where $\varphi_{1_{|}n=4}$ and $\varphi_{2_{|}n=3}$.

The *SGPNN* introduces the multi activation function Neuron (*MAFN*) to address the architecture limitation of the *PNN* to rank different lengths of output layers. The *MAFN* contains the same number of inputs because they share the same $w_{m}$ weights with input neurons where $w_{m}$ is the weight of middle layer, $y_{in}=\sum a_{i}\cdot w_{i}$. *MAFN* contains *k* number of φ activation function and lr learning rate, k=n, where *n* is the number of output layer. For example, Figure 6 shows a *MAFN* which has two φ, where each function has a single output; It is graphically represented by multiple #*n* output links because *PN* connects only to *n* number of output neurons where S=n+1 and *s* is φ step number.

As shown in Figure 6, $\varphi_{1_{|}n=4}$ and $\varphi_{2_{|}n=3}$ of the *MAFN* are connected to 2 output groups of 4 and 3 neurons, respectively.

In a conventional *ANN*, the sufficient number of hidden neurons to achieve convergence is determined by the Cao and Mirchandani theorem [42]. In an *n* dimensional space, the maximum number of regions that are linearly separable into *M* regions using *h* hidden nodes is

(3) $$M\left(h,n\right)=\sum_{k=0}^{n}\left[\begin{array}{c} h\\ k \end{array}\right]\;where\left[\begin{array}{c} h\\ k \end{array}\right]=0\;when\;h<k$$

However, the *SGPNN* has multiple Euclidean n-spaces for each output layer. Therefore, m·n<$k_{mafn}$, where *n* is the n-dimensional Euclidean space and *m* is the number of spaces per each output layer.

### 2.5. SGPNN Functionality

**Figure 7:**
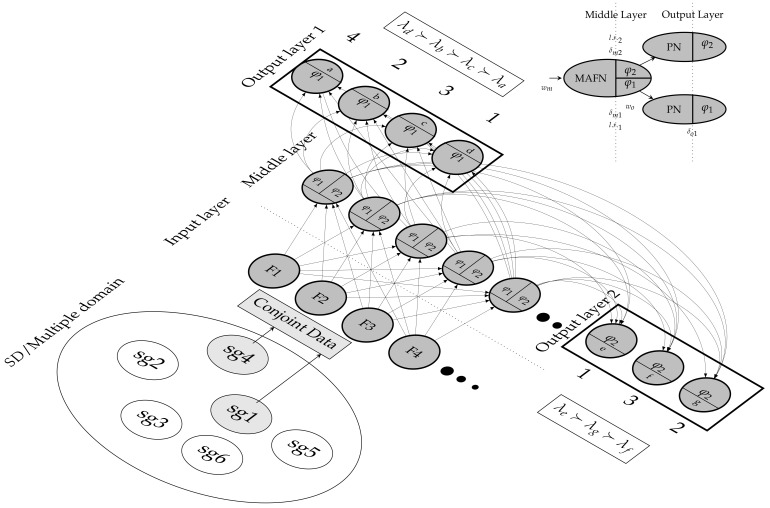
An example of Two subgroups architecture of *SGPNN* to rank conjoint data from two subgroups data, each group has 4 and 3 labels respectively, where $\varphi_{1_{n}=4}$, $\varphi_{2_{n}=1}$, $f_{in}=4$. A video demo of 2-subgroup architecture is available in [43].

**Figure 8:**
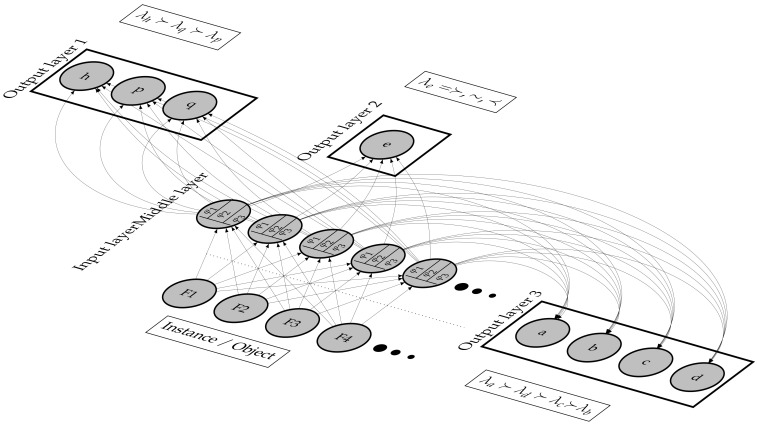
Three subgroups architecture *SGPNN* used in ranking emotions dataset where $\varphi 1_{n=4},\;\varphi 2_{n=3},\;\varphi 3_{n=3}$, and $f_{in}=4$. the second subgroup is represented by one node that has 3 values (1, 2, and 3) mapped to preference relations $\lambda_{e}=≻,∼,≺$.

The *SGPNN* is designed to address the architectural shortcoming of *PNN*s not being extendable by ranking label’s groups separately. The *SGPNN* ranks different sizes of output layers while maintaining the single middle layer design. It has two types of neurons, *PN* and *MAFN*, which are used in the output and middle layers, respectively. The input layer represents one instance of data features. The middle layer has multiple *MAFN*s that use a separate learning rate and φ activation function for each output layer. The *SGPNN* is geometrically fully connected; however, *FF*, *BP*, and *UW* are functionally separated for each wo output layers’ weights as illustrated in Figure 7. The weights of the *MAFN* are updated by the summation of all the $\delta_{m}$ errors learning rate, $\sum_{i=1}^{k}\left(lr_{i}\cdot \delta_{mi}\right)$. Each output layer is a group of *PN*s that represent the ranked labels. The *SGPNN* scales up by increasing the number of *MAFN*s. Figure 8 illustrates examples of three subgroups architecture used for ranking emotions dataset where the first, second, and third group has 3, 1, and 4 labels respectively, to solve the problem π=(*h*≻*p*≻*q*)⊥(*e*)⊥(*a*≻*b*≻*c*≻*d*). The second subgroup has one label *e* that has three ranking values (1, 2, 3), which represent the preference values (≻,⊥,≺) between the two subgroups. The learning of the ranking process is executed in three steps; *FF*, *BP*, and *UW*. The learning stops after 20,000 epochs or Spearman’s ρ reaches 1. A video demo that shows the ranking learning process using simple toy data are available at [43].

## 3. Data Preparation and Learning Algorithm

This section describes data combination, the ranking unification preprocessing and *SGPNN* learning steps (*FF*, *BP* and *UW*).

### 3.1. Conjoint Data

The Dataset is synthesized by concatenating the features and multiply the data point for each subgroup as shown in Equation (4).

(4) $$F_{sum}=\sum_{i=1}^{ns}F_{i}\;,\;D_{sum}=\prod_{i=1}^{ns}D_{i}$$

where $F_{i}$ number of features per dataset *i*, ns is number of dataset and $D_{i}$ is number of data instance per dataset *i*.

### 3.2. Ranking Unification

We introduce a new method for creating label ranking ground truth by converting the unrestricted ranking to restricted ranking by unifying the data instances and adding subgroups to the labels. The percentage of a unique ranking is measured using Equation (5).

(5) $$U_{\pi }=\frac{numberofdistinct\;rankings}{n}$$

The number of subgroups is determined by the maximum number of repeated records using Equation (6)

(6) $$sg=Max(x_{r})$$

where sg is the number of subgroups and $x_{r}$ is the number of duplicated data records. This paper applies Algorithm 1 to convert the data from non-restricted rankings with no ground truth to unique groups of label ranking by removing duplicated data instances and accumulating the corresponding labels in a subgroup. The algorithm removes the duplication and assigns the corresponding labels as a subgroup to one unique data record. For non-repeated records, the additional subgroup has values of zero.

**Algorithm 1** Ranking Unification

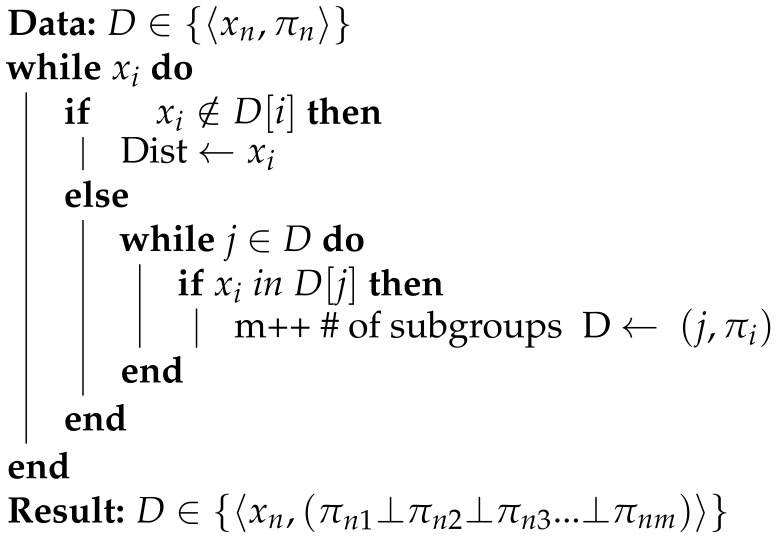



### 3.3. SGPNN Learning Steps

This section shows the *FF*, *BP* and *UW* processes in the middle and output layer of the *SGPNN*.

#### 3.3.1. Middle Layer *FF*

The output of single *MAFN* connected to subgroup *j* is shown in Equation (7)

(7) $${\left.Y_{j}=\varphi_{j}\sum_{i=1}^{d}x_{i}\cdot wm_{i}\right|}_{j=0}^{g}$$

where *g* is the number of subgroups, $wm_{i}$ is the weight of the middle layer of *MAFN* index *i*, *x* is the input value of MAFN, *d* is the number of input features, and $\varphi_{j}$ is the activation function per subgroup.

#### 3.3.2. Output Layer *FF*

The output of single neuron in subgroup *j* is shown in Equation (8)

(8) $${\left.Y_{j}=\varphi_{j}\sum_{i=1}^{m}x_{ij}\cdot wo_{ij}\right|}_{j=0}^{g}$$

where *m* is the number of *MAFN*s connected to subgroup *j* and $wo_{ij}$ is the weight of output layer of subgroup *j* and *MAFN* index *i*.

#### 3.3.3. Output Layer *BP*

The output error $\delta_{oj}$ of a single output neuron per subgroup *j* is given in Equation (9) where Error is the differentiation of Spearman correlation and activation function.

(9) $$Err_{j}=\rho_{j}ʹ=\frac{-6\cdot \sum_{k=1}^{o}\left(2yt_{k}-y_{k}\right)}{n(n^{2}-1)}\;,\;\delta_{oj}=\rho_{j}ʹ\cdot \varphi_{i}ʹ$$

$\varphi_{j}$ is *SS* function per subgroup from Equation (1).

(10) $$\varphi_{j}=-\frac{1}{2}\cdot \left(\sum_{s=0}^{n}\tanh\left(\frac{-100}{b}\cdot y_{o}+c\left(1-\frac{2s}{n-1}\right)\right)\right)\;+\;\frac{n}{2}$$

where $\delta_{oj}$ is the error of output neuron and *n* is number of labels in subgroup *j*.

The $\delta_{oj}$ in Equation (11) is obtained by differentiating of Equation (10) and substituting the result into Equation (9)

(11) $${\left.\delta_{oj}=(\frac{-6\cdot \sum_{j=1}^{o}\left(2yt_{j}-y_{i}\right)}{n(n^{2}-1)})\cdot \varphi_{j}ʹ\right|}_{j=0}^{g}$$

(12) $$\begin{array}{cc} \varphi_{j}\prime & =(-\frac{1}{2}\cdot (\sum_{s=0}^{n}1-\tanh{\left(\frac{-100}{b}\cdot y_{o}+c\left(1-\frac{2s}{n-1}\right)\right)}^{2}\cdot \frac{-100}{b}\cdot y_{o}\\ & +(\frac{-100}{b}\cdot \tanh\left(\frac{-100}{b}\cdot y_{o}+c\left(1-\frac{2s}{n-1}\right)\right)))) \end{array}$$

#### 3.3.4. Middle Layer *BP*

The output error $\delta_{m}$ is calculated in Equation (13).

(13) $${\left.{Err}_{j}=\sum_{i=0}^{o}wo_{ij}\cdot \delta_{o_{ij}}\right|}_{j=0}^{g}\;,\;{\left.\delta_{mj}=Err_{j}\cdot \varphi_{j}ʹ\right|}_{j=0}^{g}$$

Then after substitution of $\varphi_{j}\prime$, $\delta_{m}$ *MAFN*’s error in Equation (14).

(14) $$\begin{array}{cc} \delta_{mj} & =Err_{ij}\cdot -\frac{1}{2}\cdot (\sum_{i=0}^{n}1-\tanh{\left(\frac{-100}{b}\cdot x+c\left(1-\frac{2i}{n-1}\right)\right)}^{2}\\ \; & \cdot \frac{-100}{b}\cdot x+\left(\frac{-100}{b}\cdot \tanh\left(\frac{-100}{b}\cdot x+c\left(1-\frac{2i}{n-1}\right)\right)\right)) \end{array}$$

#### 3.3.5. Output Layer UW

The process to update the weights using gradient ascent with sums of $\delta_{o}$ is shown in Equation (15)

(15) $${\left.\sum_{i=1}^{m}wo_{ij_{|}new}=wo_{ij_{|}old}+\left(lr_{j}\cdot \delta_{o_{ij}}\cdot y_{ij}\right)\right|}_{j=0}^{g}$$

where $lr_{j}$ is the learning rate for subgroup *j* and $y_{ij}$ is the input multiply by wo from middle layer of index *i* of *MAFN* to the subgroup *j*.

#### 3.3.6. Middle Layer UW

Updating the weights of the middle layer is shown in Equation (16)

(16) $${\left.\sum_{i=1}^{d}\left(wm_{ij_{|}new}=wm_{ij_{|}old}+lr_{j}\cdot \delta_{m_{ij}}\cdot y_{i}\right)\right|}_{j=0}^{g}$$

where $y_{i}$ is the input multiply by wm from input layer of index *i* of input neuron.

### 3.4. Dropout Regularization

We apply dropout as a regularization approach to enhance the *SGPNN* validation performance to reduce over-fitting using 50% probability. The process assigns a random number from −0.9 to 0.9 and stop using the weights with less than 0.5 of the random value per iteration for wo and wm.

## 4. Experiments

### 4.1. Datasets

The *SGPNN* is experimented on both real-world and semi-synthesized (*s-s*)/conjoint datasets. The real data have multi-label subgroups for one set of features, e.g., restaurant-food-services. The *s-s* data are collected from different domains. The features from the same domain have small variations, e.g., the German elections dataset has examples of a relevant subgroup where features are collected from the same context. We examined the data uncertainty by measuring the percentage of $U_{\pi }$ unique multi-label ranking. Given that *d* is the amount of the data, The description is presented in Table 2.

#### 4.1.1. Restaurants Rating

The restaurant-food-services dataset is built using actual food quality and customer service reviews from the recommender systems domain [30] and contains multi-label subgroups. The features of this dataset are customer profiles and geographical location. The two subgroups are food quality and customer service, and each subgroup has 130 multi-label, representing the number of restaurants. To simplify the calculation, we use part of the data containing 5, 10, and 20 restaurants for the two groups in three small datasets and select the corresponding features records of users’ profiles who rated these restaurants.

#### 4.1.2. German Election in 2005 and 2009

The german-2005/9 is an *s-s* conjoint dataset from two real datasets based on German election in 2005 and 2009 [31,32]. The multi-label of the two datasets is grouped into two label subgroups. However, the 2009 data used features to rank both 2005/9 labels because 2009 features have historical data and user profiles for the 2005 election.

#### 4.1.3. Emotions

The emotions dataset is used for subgroup preference relations(≻,∼,≺). The original Emotion dataset is used to detect six types of emotions based on listing to different type of music where the music belongs to many to one or many emotion types. The original dataset has six classes (amazed/surprised, happy/pleased, relaxing/calm, quiet/still, sad/lonely, angry/fearful). The data are modified by creating two subgroups. Music reflects both Positive feelings for (amazed-surprised, happy-pleased, relaxing-calm, quiet-still) and the Negative feelings for (sad-lonely, angry-fearful) [44]. Table 3 shows the heuristic rules applied for the preference relation between positive and negative feeling subgroups based on the subgroup labels’ ranking. The ranking of sub-labels starts from 1 to 3. 1–3 represents the ranked value from 1 to 3.

#### 4.1.4. Irrelevant Subgroups Data

We create a new hypothetical conjoint dataset from three different domains (biology, chemistry, and trades) for preference mining analysis to study data similarity and measure the *SGPNN* performance against other ranking approach. The conjoint data are collected from the benchmark and well-known multi-label ranking datasets from different domains specifically; iris, wine, and stock [26] to compare the performance of these data as subgroups with previous approaches that experimented with those datasets as a single problem.

#### 4.1.5. Label Ranking Benchmark Dataset

The sushi [33,34] is a multi-label the dataset that has an unrestricted multi-label ranking as some identical data features have different multi-label rankings. The unrestricted ranking is converted into a restricted subgroup of multi-label for each instance of the data by removing the duplicated features and assign the labels for each repeated instance as a subgroup to a unique feature. Creating unique instances reduces the number of instances from 5000 to 4825 instances. Therefore, the maximum number of repeated instances is three, which means that the dataset has three subgroups. The instances that have unique second or third subgroups have zeros values.

### 4.2. Results

**Figure 9:**
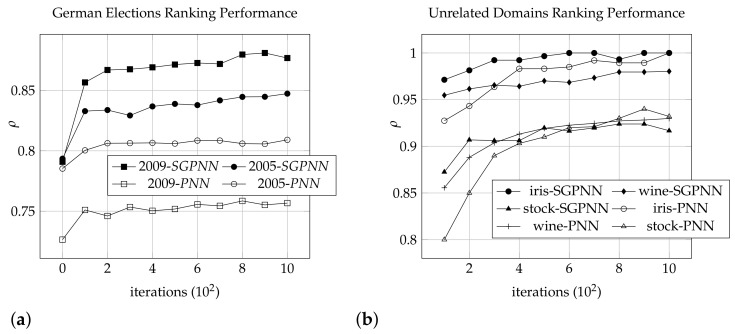
Training convergence of *PNN* and *SGPNN* using (**a**) german election 2005 and 2009 dataset. (**b**) iris, wine and stock dataset.

For the experiments, the datasets are divided randomly into the ratio of 80:20, 80% for training and validation and rest 20% for testing. Further 5-fold cross validation is adopted for 80% of training and validation to reduce the variance due to creation of data from different sources. We use sequential search by saving the best results’ hyperparameters after five-fold cross-validation. The hyperparameters are the scale factor from −b to *b*, where *b* is the *SS* boundary value, learning rate, and the number of iterations is 1000 epochs and learning rate. the validation is reduced to two-fold cross validation for unrelated data to reduce the variance, i.e., wine-iris-stock. This configuration is used for evaluating both the *PNN* and the *SGPNN*. The results are presented in Table 4. The ranking convergence of training data of the 2005 and 2009 German elections are illustrated in Figure 9a. The figure shows the ranking performance of conjoint data using *SGPNN* outperforms the ranking of 2005 and 2009 datasets separately using *PNN*. Table 4 shows the testing results of the models after 5000 epochs. We compare the single ranking *PNN*, and *SGPNN* with other multi-label ranking for iris-wine-stock dataset in terms of Kendall’s τ in Table 5. The *SGPNN* results are the ranking of each dataset as a subgroup with the other two datasets.

#### 4.2.1. Relevant Subgroup Data

The convergence of training data of the 2005 and 2009 German elections are illustrated in Figure 9a by subgroup and separate datasets, where the training model ranks convergence in terms of Spearman’s ρ and the number of iterations. It is noticed that *SGPNN* outperforms both different ranks of the 2005 and 2009 datasets using the *PNN*. The validated models’ testing results of the best epoch’s hyper-parameters are displayed in Table 4.

#### 4.2.2. Non-Relevant Subgroup Data

The results of the training data of conjoint iris, wine, and stock are illustrated in Figure 9b by *SGPNN* comparing to ranking them separately using *PNN*, in additional to the state-of-the-art methods of testing data as shown in Table 5. It is noticed that *SGPNN* outperforms the other label ranking methods; supervised clustering [27], supervised decision tree [26], multilayer perceptron label ranking [29], and label ranking tree forest (*LRF*) [45] that rank iris, wine, and stock, respectively. Ranking the three datasets (wine-iris-stock) together gives a higher ranking than even ranking every two datasets (wine-iris), (iris-stock), or (wine-stock) using the same hyperparameters as shown in Table 4.

## 5. Discussion

### 5.1. Ranking Enhancement

The results show that learning the labels as a subgroup from a relevant domain enhances each group’s ranking compared to ranking them separately. This enhancement in ranking is almost due to sharing the network weights of two or more problems. The sharing weights accelerate the convergence, similar to reinforcement learning. This paper proposes a novel learning method to rank multi-label subgroups to support the analysis of *SD*. This approach is a part of the broader sphere of reinforcement learning to learn from multiple data sources and build a conjoint unified learning model. The computation time may increase by increasing the number of subgroups and higher rank accuracy; however, *SGPNN* deliver a unified ranking model with a higher convergence rate and high testing accuracy.

### 5.2. Convergence Fluctuation

The dataset wine-stock and iris-stock take a longer time for convergence due to data separability and complexity; thus, convergence for each group of labels is not linear. This non-linearity creates fluctuations more than the ranking of a single label group. These fluctuations are not related to the gradient error in ranking, but it is the average ranking between two subgroups as each subgroup tends to increase the ranking, it updates its weights which reflect on the shared weights, which may reduce the convergence of the second group. The fluctuation is shown in the video link of convergence of two groups using toy dataset [43]. The convergence fluctuations are not noticed when we use three subgroups together, i.e., the iris-wine-stock dataset using the same hyper-parameters of two subgroups *SGPNN*.

### 5.3. Potential Applications

*SGPNN* could be used in many potential applications, i.e., brain-computer interface (*BCI*) applications where EEG data may have ambiguity, complicated, and unbalanced. Another medical application is where data fusion is collected from different sensors, i.e., the study of human emotions recognition. *SGPNN* could be part of an expert system to build accumulated learning model for judgment, elections, medical diagnosing from different conjoint historical data.

## 6. Conclusions and Future Works

The *SGPNN* is a new step in preference learning to predict the subgroups from conjoint data by proposing a simple three layers *FF* network that has different outputs to build the conjoint model from a different group of data. This paper introduces a simple network with one middle layer and a new activation function to speed up the learning to rank using the new Spearman objective function. This paper introduces the novel *MAFN* to serve more than one group of labels. In addition, creating conjoint data from multiple datasets reinforce the learning to rank and enhance accuracy. The proposed network with one middle layer simplifies the process of *FF*, *BB* and *UW* in three steps for middle and output layer comparing to the conventional *ANN*.

The future work of *SGPNN* is to coupling the relation with different *SD* methodologies to rank the subgroup. The data used in the experiment are relatively tiny; thus *SGPNN* opens a road to develop a deep learning network based on *MAFN*, *PNN*, Spearman error function, and *SS* function to accelerate the learning to build a more complicated conjoint model. The *SGPNN* integrates with *SD* to study the relations, similarity, and separability from different domains to have a shared learning model.

## Figures and Tables

**Table 1:** Comparison between classical *FF-ANN* and *PNN*.

Type	*FF-ANN*	*PNN*
Input layer	one feature/instance	one instance
Hidden layer	one/multilayer	single layer
Activation function	conventional functions *	*SS*
Gradient	descent	ascent
Objective function	*rms*	*spearman* ρ

* relu, logistic, sigmoid, tanh, gaussian, softmax, maxout.

**Table 2:** Datasets description used for *SGPNN* evaluation.

Dataset	Category	Domain	Type	Sub. Rel.	Inst.	Attr.	Sub.	Labels	$U_{\pi }$
rest-food-services	user rating	single	real	∼	92	13	2	5, 5	87.7%
					100	13	2	10, 10	76.9%
					176	13	2	20, 20	57.7%
german-2005/9	election	single	s-s	∼	412	31	2	5, 5	100%
emotions	music			≻,∼,≺	392	72	3	4, 2	100%
sushi	user rating			⊥	4825	10	3	10, 10, 10	95%
iris-wine	bio.-chem.	multi.	s-s	⊥	26,700	17	2	3, 3	99.7%
iris-stock	bio.-trades				142,500	9	2	3, 5	99.8%
wine-stock	chem.-trades				169,100	18	2	3, 5	100%
iris-wine-stock	bio.-chem.-trades				25,365,000	22	3	3, 3, 5	99.9%

**Table 3:** The relation between positive and negative emotional subgroups.

Sub1.			Sub2.	Sub3.	
**Positive Feeling Sub.**			**Rel.**	**Negative Feeling Sub.**	
**Amazed**	**Happy**	**Relaxing**		**Sad**	**Angry**
**Surprised**	**Pleased**	**Calm**		**Lonely**	**Aggressive**
1	1–3	1–3	≻	1–3	1–3
1	1–3	1–3	∼	1–3	1
2 or 3	1–3	1–3	≺	1–3	1
2 or 3	1–3	1–3	∼	1–3	1–3
2 or 3	1	1–3	≺	1	1–3
2 or 3	2 or 3	1–3	≻	1	1

**Table 4:** Performance comparison of *SGPNN* and *PNN* on conjoint and other dataset.

Dataset	S. Group	Scale	#MAFN	L.r.	*PNN*	*SGPNN*
rest-food-serv.	food quality	−1:1	100	0.06	0.814	0.912
	customer service			0.07	0.898	0.902
german election	year 2005	−20:20	100	0.05	0.8125	0.897
	year 2007			0.06	0.762	0.821
emotions	positive feeling	−10:10	100	0.05	0.616	0.87
	negative feeling				0.56	0.82
sushi	unique user pref. 1	−20:20	100	0.05	0.741	0.851
	unique user pref. 2					0.813
	unique user pref. 3					0.92
iris-wine	biology (iris)	−10:10	200	0.0007	0.917	0.933
	chemistry (wine)				0.901	0.804
iris-stock	biology (iris)	−10:10	200	0.0007	0.917	0.91
	trades (stock)				0.834	0.75
wine-stock	chemistry (wine)	−10:10	200	0.0007	0.901	0.911
	trades (stock)				0.834	0.732
iris-wine-stock	biology (iris)	−10:10	200	0.0007	0.917	0.912
	chemistry (wine)				0.901	0.856
	trades (stock)				0.834	0.956
Average					0.82	0.865

**Table 5:** Performance comparison of *SGPNN*, *PNN* and state-of-the-art label ranking approaches.

Multi Label Ranking Methods						
Dataset	S. Clust.	*DT*	*MLP-LR*	*LRF*	*PNN*	*SGPNN* (Iris-Wine-Stock)
iris	0.814	0.966 (IBLR)	0.925 (LA)	0.947	0.917	0.921
wine	0.898	0.949 (IBLR)	0.931 (LA)	0.882	0.901	0.865
stock	0.566	0.927 (IBLR)	0.745 (CA)	0.895	0.834	0.956
Average	0.6	0.848	0.692	0.730	0.884	0.914

## Data Availability

The data presented in this study are openly available in [43].
